# Fighting for Olympic dreams and life beyond: Olympian judokas on striving for glory and tackling post-athletic challenges

**DOI:** 10.3389/fpsyg.2023.1269174

**Published:** 2023-09-26

**Authors:** Hee Jung Hong, Seung Han Hong

**Affiliations:** ^1^Faculty of Health Sciences and Sport, University of Stirling, Stirling, United Kingdom; ^2^Department of Sport Coaching, Korea National Sport University, Seoul, Republic of Korea

**Keywords:** athletes’ career development, athletes’ career transition, judo, Olympic Games, pre-retirement planning

## Abstract

**Objectives:**

This study explores the experiences of Olympian judokas, examining both their pursuit of excellence to compete at the Olympics and their subsequent transition out of judo. The aim is to offer empirical evidence regarding the challenges they face in realizing their Olympic dreams, and to shed light on the transitional challenges, available resources, and needs they face as they move toward post-athletic lives.

**Methods:**

We conducted semi-structured interviews with eight Olympian judokas: five males and three females, all of whom have retired from competitive judo. These participants are from Portugal (*n* = 1), Republic of Korea (*n* = 2), and the United Kingdom (*n* = 5). We employed thematic analysis, which led to the identification of five main themes: (a) From Dreams to Olympic Reality, (b) Facing the Void: Loss of Goals and Identity, (c) The Crucial Role of Social Support, (d) Dual Aspects of Pre-Retirement Planning, and (e) The Double Edge of Organizational Support.

**Findings:**

The findings highlight the significant challenges faced by Olympian judokas, including goal and identity loss post-retirement, and the need for comprehensive and accessible organizational support, particularly psychological assistance, to assist in their transition to post-athletic life.

**Implications:**

The findings not only enhance our understanding of judokas’ experiences during transition but also offer insights that could guide the development of tailored support programs. It is critical for sport governing bodies and practitioners to apply these insights in creating comprehensive and easily accessible support systems, which will ensure a smoother transition to post-athletic life for high-performance athletes.

## 1. Introduction

Achieving and perhaps even standing out at the Olympic Games is perceived by a number of high-performance athletes as the peak of their sports careers. This has led scholars to emphasize the role of psychological readiness and assistance in achieving Olympic success (Wylleman et al., 2009; Wylleman and Rosier, 2016). High performance athletes consistently push their physiological boundaries and challenge their physical capacity to realize their Olympic aspirations. During their prime, their accomplishments are recognized and celebrated as they stablish what appear to be unattainable physical standards (Silver, 2021). However, the rigorous requirements of top-tier sports can sometimes hinder athletes from exploring diverse interests early in life (Lavallee, 2005; Stambulova et al., 2007; Barker-Ruchti et al., 2019). As a result, many of these athletes often face significant challenges in their post-athletic life, such as finding a new job, seeking social support from trusted individuals, and experiencing social isolation (Lavallee, 2006; Warriner and Lavallee, 2008; Park et al., 2013; Martin et al., 2014). Researchers reported that involuntary retirement can be significantly related to transitional issues (Wylleman and Lavallee, 2004; Martin et al., 2014; Stambulova et al., 2021). More importantly, such transitional issues can be associated with mental health problems such as eating disorders, substance abuse, and tragically, even instances of suicide (Jones et al., 2005; Malcolm and Scott, 2012; Mannes et al., 2019).

The journey of high-performance athletes after they step away from their sport has gained increasing attention from both researchers and the broader public. Initial studies on athletes ending their competitive careers can be traced back to the late 1960s, with Mihovilovic’s pioneering efforts (Mihovilovic, 1968). Through the 1980s, there was an increase of studies examining aspects like athletes’ career progressions and the eventual shift toward retirement (Stambulova et al., 2009). In a more recent review by Stambulova et al. (2021), it was noted that early studies, spanning the 1960s to the 1980s, often tackled athletes’ retirements from a non-sport specific framework. The 1990s, however, marked a transition to a more holistic approach athletes’ entire careers and within career transitions (e.g., junior to senior transition) using sport-specific frameworks. In contemporary times, this lens has expanded further, considering athletes within the broader background of their cultural and social ecosystems. While life shifts are known stress triggers requiring adaptation (Brissette et al., 2002), the act of leaving a sport is especially transformative and could influence an athlete’s psychological well-being (Lavallee, 2005; Park et al., 2013). Notably, top-tier athletes are prone to a spectrum of mental health challenges (Rice et al., 2016), which could appear as anxiety, depression (Gouttebarge et al., 2016; Schuring et al., 2017), or, in severe cases, even lead to suicides (Hong, 2018). Using well-regarded theoretical background (Taylor et al., 2005; Stambulova et al., 2009), Park et al. (2013) highlighted 15 critical determinants influencing the quality of an athlete’s shift away from their sport. Their exhaustive list ranged from aspects like an athlete’s self-identity to financial stability (see Park et al., 2013 for further detail). Even with such comprehensive insights, there is still room to explore further. Specifically, Park et al. (2013) emphasized the importance of pre-retirement planning for ensuring a smooth and successful transition. However, the strategies elite athletes adopt for such preparations, or the reasons some may overlook this step, remain under-researched and require additional investigation.

In a similar vein, Stambulova et al. (2009) highlighted several crucial elements of the athletic transition process. They pinpointed a variety of factors leading to the end of sports careers and noted the individual differences in reactions to such terminations. Particularly significant was an athlete’s sense of having a choice in their transitioning out of sport and how it impacted their adaptation to post-athletic life. They also emphasized the importance of athletes beginning preparations for life after their sports career early on. In addition, the resources accessible to these athletes and the fact that about 15–20% of elite athletes seek psychological help after retiring were highlighted. As a result, these researchers stressed the value of early retirement planning, the autonomy in making the decision to retire, developing various personal identities, having access to support networks, and the development of proactive coping strategies as key to aiding athletes’ adaptation to life after sport. Since the key to an athlete’s successful transition lies in their ability to adapt, and our study investigates these challenges and needs, aiming to improve support for high performance athletes during this significant phase.

**FIGURE 1:**
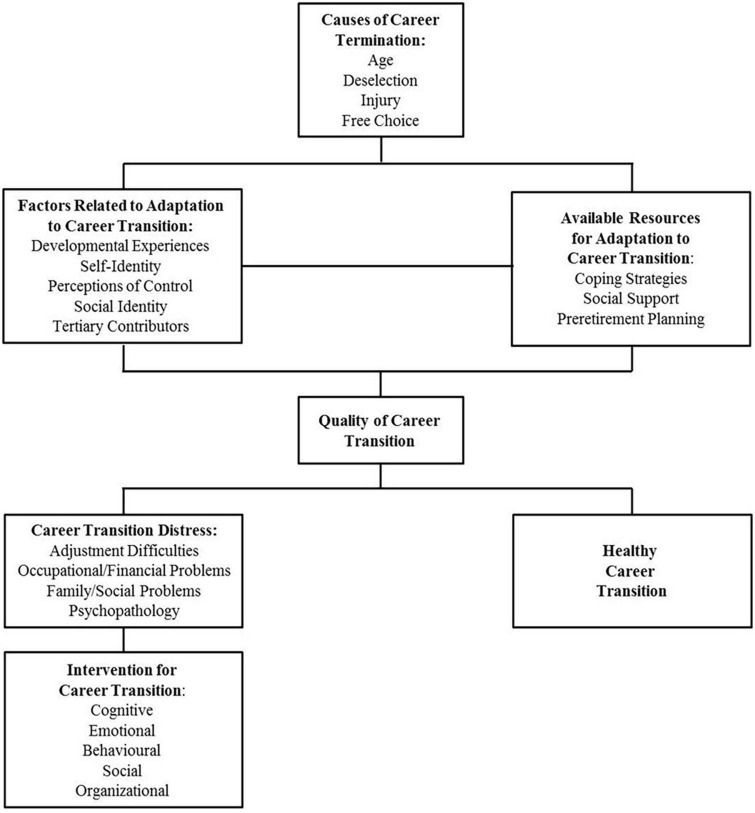
Conceptual Model of Adaptation to Career Transition (Lavallee et al., 2014).

High performance will all eventually face retirement, though the timing and circumstances of their career termination can be unpredictable. Retirement is not just a career change but brings about challenges related to self-identity and coping (Stambulova et al., 2009, 2021). Many high-performance athletes, though, find themselves unprepared for transitioning out of sport, lacking resources for a smooth career shift, leaving them vulnerable to a destressing transition (Hong and Coffee, 2018). In our endeavor to understand the journey of retired Olympic judokas, we have employed the Conceptual Model of Adaptation to Career Transition (Lavallee et al., 2014; see in Figure 1) as the foundational framework of our research.

The Conceptual Model of Adaptation to Career Transition was initially developed based on research on athletes’ transitions by Taylor and Ogilvie (1994) has been modified by Lavallee et al. (2014). This model offers insights into different aspect, emphasizing how sports psychologists and other experts might best support athletes during these transformative phases. The model outlines four primary triggers for ending an athletic career: aging, deselection, injuries, and personal choice. However, while these reasons provide a baseline, it is important to recognize and understand additional factors that could influence an athlete’s decision to retire. For instance, Hong et al. (2020) investigated support systems and interventions available globally for athletes facing career transitions due to anti-doping policy violation. Their findings demonstrated that doping sanctions could significantly affect athletes’ retirement. This model also points to five key elements influencing an athlete’s adjustment to a new career phase. Past studies indicate that high performance athletes, during this shift, might struggle with identity crises. This is attributed to the restricted chances they get to develop diverse identities during their sporting commitments (Lally, 2007; Park et al., 2013).

As highlighted earlier, for athletes managing career transitions, it is critical to identify resources such as coping mechanisms, social support network, and pre-retirement planning. When the pressures of such transitions surpass these resources, athletes can struggle with challenges spanning from work-related and financial issues to family disputes and mental health complications (Stambulova et al., 2009, 2021). In these instances, they may benefit from interventions targeting cognitive, emotional, behavioral, and organizational aspects (Lavallee et al., 2014). Previous studies highlight the significant role played by sport governing body in implementing support schemes/programs as a form of organizational interventions (Surujlal, 2016; Hong and Coffee, 2018). Some research, including findings from Hong and Coffee (2018) and Torregrossa et al. (2020), demonstrates the presence of career support systems tailored for athletes. Specifically, Hong and Coffee identified these programs in 19 countries, indicating the proactive steps taken by sport governing bodies and organizations to support athletes during these critical shifts. However, there is a noticeable gap in research when it comes to understanding how athletes engage with these organizational supports during their transitions. Thus, the present study aims to explore the experiences of Olympian judokas striving to compete at the Olympics. By examining their transition out of judo, this research intends to provide empirical evidence about what it takes to achieve their Olympic dreams, the challenges they face during transitions, the resources available to them, and their needs after their athletic careers.

## 2. Materials and methods

### 2.1. Design

This research employed a case study design to understand the journey of Olympian judokas, from their dedication to competing at the Olympics to their subsequent transition out of the sport. The method allowed for a close examination of this unique scenario, emphasizing the personal stories of the involved individuals. Since the aim was to identify the perspectives of these participants concerning their journey (Smith and Eatough, 2019), the research is positioned in an interpretivist paradigm (Mallett and Tinning, 2014), which is guided by a relativist ontology along with a subjectivist epistemology. This empowers the researchers to identify how individuals interpret their own experiences (Mallett and Tinning, 2014; Smith and Eatough, 2019). Phenomenological research in the interpretive realm strives to explain, understand, and shed light on specific phenomena (Tuohy et al., 2013), essentially identifying the core of the actual lived experiences (Creswell, 2007). As Merriam and Greiner (2019) state, this research method offers an avenue to examine internal experiences that often remain unexplored in our daily lives.

To explore the personal stories of the participants, we used semi-structured interviews, ensuring a comprehensive capture of their narratives (McArdle et al., 2012). Recognizing that all participants had a mutual experience and situation, attention was paid to the significance each individual assigned to their journey (Creswell, 2007). Given that all participants were engaged in the collective experience of Olympic competition and its subsequent transition, this approach was deemed most suitable for our investigation.

### 2.2. Participants

We recruited eight participants in our study, five of whom were males and three were females, all retired from competitive judo. During the data collection phase, the participants’ ages ranged between 32 and 41 years, with a mean age of 35.50 (SD = 3.20); years after retirement are between 1 and 13 years (*M* = 5.25, SD = 4.05). Detailed participant information is presented in Table 1.

**TABLE 1:** Participant information.

Participants	Gender	Age	Nationality	Years after retirement
Olympian 1	Male	Early 30s	Republic of Korea	5
Olympian 2	Male	Early 40s	Republic of Korea	13
Olympian 3	Male	Early 40s	United Kingdom	7
Olympian 4	Male	Mid 30s	United Kingdom	1
Olympian 5	Female	Early 30s	United Kingdom	5
Olympian 6	Female	Early 30s	United Kingdom	1
Olympian 7	Female	Mid 30s	United Kingdom	1
Olympian 8	Male	Late 30s	Portugal	9

We have employed broader terms concerning the participants’ age as an additional measure to secure their identities.

### 2.3. Data collection

Following institutional ethical approval, both author’s contacts were used to recruit a purposive sample (Noy, 2008). Taking into account the availability and preferences of participants during the data collection, five face-to-face interviews were conducted while three others were facilitated through video calls, using platforms such as WhatsApp and Microsoft Teams. Prior to the interviews, participants were provided with the semi-structured interview questions. This proactive measure gave them sufficient time to peruse the questions and determine their comfort levels in responding to them, ensuring we remained ethically sensitive. To document the interviews, we used both a voice recorder and the recording feature on Microsoft Teams. Due to the semi-structured format of the interviews, participants enjoyed the latitude to discuss significant moments not explicitly covered by the predetermined questions, further enhancing the depth and quality of our findings (McArdle et al., 2012).

To ensure consistency across interviews, we developed an interview guide based on our research questions and insights from existing literature (e.g., Stambulova et al., 2009; Park et al., 2013; Lavallee et al., 2014; Torregrosa et al., 2015; Hong and Fraser, 2021). The guide addressed the sport background, prompting questions such as “When did you start your elite judo career?” and “What motivated your interest in elite judo?”. It also explored experiences related to preparing for and competing in the Olympics, with inquiries such as “How would you describe your overall preparation for the Olympics?”, “What challenges did you encounter?”, “How did you overcome these challenges?”, and “Can you share your overall experience of competing at the Olympics?”. Lastly, it touched upon experiences concerning the transition from competitive judo, asking participants “How was your experience transitioning out of competitive judo?”, “What challenges emerged?”, “How did you cope with these challenges?”, and “What type of support do you believe would be helpful?”.

Before participating in the study, each participant received an information sheet that detailed the purpose of study, methodology, potential risks, and benefits. After reviewing this information, they were asked to sign a consent form, confirming their agreement to participate. Once we received the signed consent form, we scheduled individual interviews for them. Providing participants with the information sheet and obtaining their consent was crucial to ensure they were fully informed about the study and their rights. This approach highlighted our dedication to upholding ethical research principles, such as informed consent and respecting participant autonomy. We posit that these steps ensured the credibility and integrity of our study. Interview durations ranged from 61 to 143 min, with an average time of 89.63 min (SD = 29.94). Each interview was transcribed verbatim. For confidentiality, we replaced participants’ names with codes such as Olympian 1, Olympian 2, and so on (see Table 1).

### 2.4. Data analysis and rigor

In this study, the authors employed the thematic analysis approach outlined in six steps by Braun and Clarke (2006) to identify critical patterns in the collected data. The method, consistent with the guidelines proposed by Braun et al. (2016), aided in highlighting key themes and patterns. To better understand the data, the authors thoroughly went through the transcripts and revisited the recorded interviews multiple times. They made notes on their preliminary observations concerning participants’ Olympic experiences and their transitioning out of sport. To ensure the results were both valid and consistent, the authors convened for three separate discussions, during which they examined the preliminary codes and overarching themes that had been identified. These conversations, held either over video or phone, were instrumental in refining the themes, ensuring there was a mutual agreement on what had been identified. For clarity and transparency, each identified theme was given a specific label and description. This thorough data analysis method was chosen by the researchers to provide a reliable narrative of the participants’ experience, reinforcing confidence in the results. Additionally, they reviewed and implemented Braun and Clarke’s (2006) 15-point checklist to ensure the quality of their thematic analysis across all six phases.

In qualitative research, it is critical to ensure our work meets rigor and trustworthiness stands. To achieve this in our study, we took several steps. Firstly, our team regularly met to review our data analysis process and the themes we identified. These discussions made sure our analysis matched what we aimed to investigate. Then, following advice from Brown et al. (2018), we kept a detailed record of the steps we took in our analysis, explaining the methods we used and why we chose them. This aligns with Finfgeld-Connett’s (2014) suggestions. Finally, to make sure our findings were sound and trustworthy, we each reviewed the analysis independently. We shared feedback with each other, similar to the “critical friends” idea from Marshall and Rossman (2006). Through these steps, we made sure our results were trustworthy and met the standards expected in qualitative research.

## 3. Results

Five themes were identified from the thematic analysis (for further details, see Table 2).

**TABLE 2:** Themes identified by thematic analysis.

Theme	Sub-themes
From dreams to Olympic reality	Strong drive and motivation Persevering with pursuit of excellence
Facing the void: loss of goals and identity	Loss of goals Loss of identity
The crucial role of social support	Support from parents Support from coaches Support from partners and peers
Dual aspects of pre-retirement planning	Athletic commitments versus future preparations The consequences of deferred planning
The double edge of organizational support	Organizational support: encouraging athletic aspirations Organizational support subject to politics and agendas: discouraging athletic aspirations

### 3.1. From dreams to Olympic reality

All participants recalled dreaming of the Olympics from a young age, especially as they began to take judo more seriously. Each one spoke of their deep enjoyment and love for judo. This passion gave them a powerful motivation to compete at the Olympics, which they viewed as the pinnacle of their judo career. In essence, the aspiration to participate in the Olympics became their primary motivation and driving force. Olympian 8 mentioned, “I remember watching the Olympic Games in Athens in 1996. Not Athens, sorry, Atlanta. The Olympic Games have something special because you don’t go to the Olympic Games because you are rich or you are poor, pretty or not; you’re there because you are great at something. So, to be great at something, you have to put in significant effort. I realized this when I was 14 years old and decided I wanted to be one of the best in the world at judo. To achieve that, I knew I had to train harder than everyone else.” Similarly, other participants nurtured dreams of becoming top judokas in the world and competing at the Olympics. To realize these dreams, they dedicated themselves to rigorous training and consistent competitions to advance their judo careers. Despite facing a number of challenges and obstacles, they persevered in their pursuit of excellence to be among the best. Olympian 1 highlighted, “Every athlete faces moments of anxiety. I believe that this anxiety is due to a lack of confidence in one’s training. Factors like failing in one’s personal routine and management can contribute to this anxiety. Since there are things I cannot change, and I’ve trained hard with what I have, I must accept that there are things out of my control and focus on what I can do now.”

While some participants won medals, others did not. Although they recognized that simply competing at the Olympics was an honor, they could not help but look back with a sense of regret. Olympian 3 shared his experience “Between Beijing and London, I had best point in my career. But best time in my career came in the middle of the two Olympic Games. So, maybe for 18 months to 2 years in this period, I almost didn’t lose a match internationally and I was number one on the world ranking list. But it was not the right time to be an Olympic Champion.” It was worth noting that, when he was young, he left university to focus solely on judo, which was a significant decision: “I went to university when I was, I think 17… no… 18 and also started training like almost full-time judo and I was doing both, but I was loving doing judo and I didn’t enjoy my university course. I think my personality was if something is going wrong, I stick my head in the sand. So, I started doing more of judo, more, more, more, more, more and going to university less and less and less and less. […] So, I left university without doing exams in second year. At the time, I think really, I tried to cope with this on my own but not very well.”

Olympian 1 still felt frustration, “When I lost, it felt like I had been in a car accident. It was unexpected. Just as you wouldn’t anticipate getting suddenly hit by a car. When I lost, I initially didn’t want to do judo anymore. I gave it my all, and in a way, I felt hurt.” Among the participants who won a medal, Olympian 5 shared her feeling when she won a medal “I knew winning a medal wouldn’t be easy, but it happens to me. It was amazing. All my hard work paid off. […] I considered going for another Olympics, but I knew it would be very challenging.” They knew what it took to prepare for another cycle of the Olympics, and they had already invested everything they had into the event. In light of this, all participants chose to retire after the Olympics. Even though they accomplished their goals by competing in the Olympics, departing from a passion they had devoted their lives to was challenging. Olympian 8’s statements describe this sentiment effectively: “I remember, after my last fight when the competition ended, I was overwhelmed with emotion and tears. It wasn’t because I had lost; it was the realization that a journey spanning 12 years had come to an end. For over a decade, I had worked toward this, and in that moment, it felt like a door was closing.” The participants recognized and appreciated their achievements but also understood their limitations regarding continuing their athletic careers for the next Olympics. As a result, they chose to retire from competitive judo.

### 3.2. Facing the void: loss of goals and identity

Two notable shared experiences among the participants were the loss of goals and identity. Given that their primary objective was to compete at the Olympics, with all other goals directed toward a successful athletic career, they inevitably faced the issue of goal loss: “The first emotion I felt was a lack of purpose. Without a goal, there was a sense of depression. What I remember thinking back then was, just as I used to focus solely on training every single day, there was no longer a need to maintain that focus” (Olympian 1). Olympic 8 also noted, “It was a strange feeling because like I told you, I finished a cycle in my life. But at that moment I had no objective in the future. I knew that I wanted to finish university, but the university that I was studying at I didn’t enjoy that much.”

Loss of identity emerged as another central issue among the participants. Olympian 5 mentioned that she still struggled with identity issues, even though she retired 5 years prior to the data collection: “You know, I’m happy with what I’ve achieved. I have a family now… and I keep myself busy, always learning. But even so, that athletic identity of mine remains a huge part of me.” While some of them have established professions and others are exploring career options, they struggle to find interests beyond judo. Even when choosing a profession, they question whether it truly aligns with their desires: “So, the young athletes were familiar with me being like a coach, like a coach type responsibility. So, this was quite an easy transition. I think the more difficult transition was – I think partly because I left my education and then I didn’t go back to my education, I felt probably a little trapped when I was first a coach because I love judo, I liked coaching, but I wasn’t 100% sure that coaching was what I wanted to do. […] But I felt like maybe this is the only way I can make money because I have no qualification in anything else. My – all of my experience is in this. So, if I don’t want to do this, how am I going to make money? How am I going to get a job? So, I think for probably almost 2 years, I didn’t really speak to anyone about this apart from my wife.” Olympian 4, who recently transitioned to a coaching role, echoed this sentiment.

Among the coping strategies that participants employed to address the issues of goal and identity loss, two stood out: setting a new goal and seeking support. While the topic of seeking support will be explored in depth in the subsequent sub-section, participants emphasized the significance of goal setting and maintaining focus on their new objectives. Both Olympians 6 and 7 stressed the importance of concentrating on one small goal at a time, similar to how they approached judo by focusing on one fight at a time. This sentiment, shared by others, highlighted the importance of leveraging their well-developed skills in goal setting and motivation—skills transferable to areas beyond judo.

### 3.3. The crucial role of social support

The participants emphasized the importance of social support throughout both their athletic careers and their transition periods. When they began their judo journeys and faced critical decisions in their careers, parental support was crucial in enabling them to pursue their passion for judo. While parents initially had concerns about a career in athletics and preferred their children to pursue academic paths like their peers, they eventually offered their support. This encouragement was so important for the participants in pursing their dreams: “They didn’t stop me, but they were not very, very happy. They had no background as sportsmen, so they didn’t have this sport culture. So, for them the important thing was to study and to have a peaceful life in the future. Sports can be trouble here” (Olympian 8). Olympian 2 also shared such experience, “Yes, my dad would say that I had a lot of athletic talent, so I should pursue it. My mum, reluctantly and with tears, would send me off. She’d be heartbroken and often tell me, even as she was crying, ‘It’s not too late; you should study.”’

As they advanced in their careers, they also received support from coaches, partners, and peers. This support was critical for the continuation of their pursuit of dreams. For instance, “I’ve got massive support from my coaches when I felt down, checking on me, keeping in touch, it was massive help (Olympian 7).” Both Olympians 3 and 5 highlighted the significant support they received from their partners during their athletic careers as well as during their adaptation to life post-retirement: “I think to catch these people I had to focus 100% on judo. But certainly, in the last part of my career, I did like many different things. So, I think one of the most important things was when I met my wife” (Olympian 3). Olympian 6 noted, “I need to spend some quality time with my friends to relieve my stress and such. If I have a good weekend with my family and friends, who always support me, I find that my training the following week goes better.” However, Olympians 1 and 4 experienced a lack of support from their coaches, which adversely affected their performance, particularly during the Olympics and in the preparation leading up to it. For instance, Olympian 1 noted, “We were together for 4 years and were on the national team together. But I knew he wasn’t interested in me. Why? Because he never once came to me personally to teach a technique, and never once paid attention to me during training. I always trained alone, and he only coached me during competitions, even though I made it to the team on my own. Realizing I had trusted someone like this for 4 years to reach my final goal was extremely disappointing.”

While participants actively sought social support both during and after their athletic careers, they recognized that ultimately, they had to confront all challenges on their own. They believed this was because even family, coaches, partners, and friends might not fully understand the unique challenges of their careers and transitions. In addition, they felt confident in their own strength to manage these changes and demands.

### 3.4. Dual aspects of pre-retirement planning

While participants recognized the importance of pre-retirement planning during their active athletic careers, most did not manage to do so. They emphasized the challenges of creating pre-retirement plans, as athletes need to remain focused on their immediate goals and performance. Some participants pointed out that the absence of a clear point of contact for support made it difficult to even consider initiating pre-retirement planning. For instance, Olympian 2 mentioned, “I was an athlete too, but athletes mostly focus on their sports and don’t make decisions about their future careers. They think they’ll give it a try, and if it doesn’t work out, then they’ll consider what’s next. But by the time they realize it, it’s late. They’re already in their 30s. If they’d realized it in their mid-twenties, they could have done something else while still earning a salary. When they leave the sport and try to find a new path, they’re older, which makes it hard to start something new. Plus, they don’t even know where to begin or whom to ask. There might be people who’ve benefited from seeking guidance, but I haven’t seen any. There probably is some assistance if you call the association, but you have to take the initiative and knock on doors to get that help. Athletes don’t know where to start. They haven’t been educated on this. Only a few who are fortunate enough to know about such opportunities might knock on those doors.”

While none of the participants managed to establish pre-retirement planning during their athletic careers, some of the British judokas mentioned that they learned how to pursue their second career from their agencies. For instance, Olympian 3 discussed, “I worked with [the name of the agency] and then for all of the rest of my career I worked with them. So, although I was not doing like formal education, I was doing a lot of work with those guys on developing my ability to talk in front of like an audience…and also to deliver workshops to businesses around how to help businesses through the lessons from sport. They have like a tag line, like they talk about double career track, so they always talk about athletic career and okay ‘What else? What’s your second career path?’ They are always trying to speak to athletes about ‘okay it’s important that this goes well but if this goes well and you’re doing nothing with this, the rest of your life could be very difficult. So, let’s make this like double career track.”’ Some British judokas, including Olympians 5 and 6, mentioned receiving support from their sport governing bodies to cope with transitional demands and explore career options. However, they believed there should be more consistent support for transitional demands and pre-retirement planning. In contrast, Olympians 1 and 2 (from Republic of Korea) and Olympian 8 (from Portugal) expressed frustration over the lack of organizational support, feeling that their sport governing bodies neglected them once their careers ended. For instance, Olympian 8 highlighted that “If I end up having to live under a bridge afterward, they’ll just say it’s not their problem, that they’ve done their part in helping me. But I believe otherwise; it is their problem. I think there needs to be a system, rule, law – something – where governments, federations, and Olympic committees take responsibility for these individuals.”

The narratives from the participants in this section strongly suggest a need for further organizational support. This would help athletes keep a balance between pursuing excellence and preparing for life after sport, as well as assist them in adapting to post-athletic lives.

### 3.5. The double edge of organizational support

While the participants recognize the value of organizational support in fostering their athletic dreams during their careers, they also felt that such support, when influenced by the politics and agendas of sports governing bodies, could dampen their aspirations. Although organizational support can manifest in various ways – from financial and informational assistance to psychological and emotional support – the participants predominantly spoke of financial aid during their athletic career and psychological support for their life post-athletics. Regardless their nationalities, they benefited from financial support from their respective sport governing bodies, especially during periods when they were aspiring athletes, at the peak of their performance, and notably when preparing for the Olympics, which they greatly valued. However, the British judokas encountered challenges with funding cuts due to decisions made by the sport governing body. They were asked to relocate to a central training center, leaving behind their regional facilities where they had trained extensively. All the participants in this study chose not to move, believing that staying with their familiar facilities and coaching staff was critical for optimizing their training and results. This preference was overlooked, leading them to lose out on funding, which adversely impacted their training and performance. One participant even pointed out that these politics and agendas were instrumental in their decision to retire. Olympian 4 bitterly discussed, “Politics is always in the background. It’s like being on the edge of funding battles. […] They just changed the funding criteria one day, and the funding was cut down just like that. They just move the goalposts at their convenience […] It’s like gambling. Performance sport is a gamble.”

Apart from financial support issues, psychological support from the sport governing body is crucial, especially during an athlete’s retirement phase. Of the participants, only Olympian 5 received such psychological assistance, which she found invaluable both during her athletic career and immediately post-retirement. However, she emphasized the need for this support to be more consistent and easily accessible, especially when athletes struggle with psychological challenges such as identity loss upon retirement. While some athletes, such as a few British judokas, can obtain support from other sources such as agencies, as discussed by Olympian 8 in a previous section, the participants’ narratives suggest that sport governing bodies should actively address this concern.

## 4. Discussion

This study explores the experiences of Olympian judokas, both in their pursuit of excellence to compete at the Olympics and in their transition out of judo. It aims to provide empirical evidence about what it takes to realize their Olympic dreams, as well as the challenges they face in transition, available resources, and the needs they encounter in their post-athletic lives. The findings offer significant contributions to both academic literature and practical applications. From a literary perspective, this study provides empirical evidence of Olympians’ journeys to excellence and the challenges and obstacles they overcame to fulfill their Olympic aspirations. While the findings align with prior studies on the loss of athletic identity, they also bring to the forefront the issue of goal loss, which is inevitable once their ultimate objectives—such as competing or winning a medal at the Olympics—are achieved. This research further corroborates previous studies on the critical roles of social and organizational support. However, it also sheds light on the less-discussed negative perspectives athletes hold regarding organizational support. Similarly, while participants highlighted the importance of pre-retirement planning in hindsight, they also emphasized that high-performance athletes often find it challenging to establish such plans during their active years (Lavallee, 2005; Stambulova et al., 2007; Barker-Ruchti et al., 2019) unless they receive adequate support. These insights resonate with the Conceptual Model of Adaptation to Career Transition (Lavallee et al., 2014), which will be elaborated upon in subsequent sections. Practically, these findings suggest essential elements that sport governing bodies should consider when developing new support strategies or enhancing current support services, ensuring they cater effectively to both retiring and post-retirement high performance athletes.

All participants shared their journeys toward the Olympics, detailing the challenges, demands, and coping strategies they employed to achieve their goals. Beyond their passion for judo, the Olympics served as a key motivator, pushing them to persist in their endeavors. The Olympic Games symbolized the pinnacle of their athletic careers. In this respect, they invested heavily in physical preparations, such as rigorous training and injury management, as well as psychological preparations, in line with findings from Wylleman et al. (2009) and Wylleman and Rosier (2016). While all participants achieved their dream of competing in the Olympics, they recognized the inevitability of their careers ending post-Olympics. They were acutely aware of their limits and believed they had reached their peak. In this context, their retirements could be viewed as voluntary. Research suggests that voluntary retirement can facilitate a smoother transition into post-athletic life (Stambulova et al., 2009; Park et al., 2013; Lavallee et al., 2014). However, this study’s findings indicate that a voluntary retirement does not necessarily mitigate the challenges of this transition. The loss of goals and identity emerged as pressing issues, with some participants still struggling with them. Consistent with earlier research (Sinclair and Orlick, 1994; Lally, 2007; Park et al., 2013), identity loss was a primary concern. While the Conceptual Model of Adaptation to Career Transition (Lavallee et al., 2014) cites identity concerns as factors affecting career transition, this study suggests the inclusion of goal loss as an equally impactful factor. To address these challenges, participants employed goal setting and sought support. Establishing and focusing on new objectives enabled them to find direction in their post-athletic lives, often in alignment with new professional pursuits. However, it is crucial to note that while some secured professions, others were still exploring career avenues, all struggled with identifying interests outside of judo. At times, they questioned their choices, tying back to the broader issue of inadequate pre-retirement planning. Their intense commitment to their sport left little room to explore external interests. Although they acknowledged the importance of pre-retirement planning, they conceded that it might be challenging to achieve without adequate support. To developed tailored support programs that consider the specific context of Olympic judokas transitioning out of sport, it is crucial to acknowledge the impact of sports on human personality and the variations in personality traits across different combat sports disciplines; customizing support services to the specific personality traits associated with each trained sport is critical for developing effective support mechanisms as Piepiora and Witkowski (2020) suggested.

The findings highlight the critical roles of both social and organizational support in athletes’ transition out of their sport. When confronted with significant challenges and barriers, participants sought social support both during and post their athletic careers, aligning with observations from previous studies such as Brown et al. (2018). Notably, the primary sources of this support shifted as participants progressed in their careers. In the early stages, parental support was significant, but as athletes matured and advanced, they increasingly turned to partners, coaches, and peers for support. This shift is elaborated upon in Wylleman (2019), in the context of the Holistic Athletic Career model. While many participants valued the support they received from their coaches, two judokas (Olympians 1 and 4) recounted negative experiences stemming from a lack of such support. This deficiency notably impacted their performance, especially during the Olympics and the preparatory phase leading up to it. Given the well-documented influence of the coach-athlete relationship on performance, as noted by Jowett (2017), the lack of a strong bond with a coach (as in the case of Olympian 1) and frequent changes in coaching staff without involving the athlete in discussions (as experienced by Olympian 4) are matters sport governing bodies should consider when strategizing and framing support mechanisms for the Olympics. In this respect, the role of coaches extends beyond enhancing athletic performance to influencing the overall well-being of the athletes. Witkowski et al. (2016) found that that combat sport athletes, such as Polish combat sport athletes including judokas, perceived their coaches as playing a critical role in their educational process, and thus, many coaches prefer to focus on educational values rather than solely on sports achievement. Fostering educational values, as preferred by most combat sports coaches, indicates that proficient coaches can play a progressively crucial role in enhancing all dimensions of health (e.g., somatic, mental, and social) of athletes and their abilities to cope with life challenges (Witkowski et al., 2016). More importantly, while participants actively sought out social support to navigate their challenges, it is crucial to acknowledge their inherent resilience and accountability. These athletes have highly developed psychological skills throughout their training and competitive judo experiences (Sarkar, 2017).

Regarding organizational support, only two British judokas (Olympians 5 and 6) mentioned receiving any from their sport governing body to cope with transitional demands and explore career options. However, it is important to highlight that such support tends to be sporadic and even temporary. Those who did experience such support emphasized the need for more consistent and regular assistance for transitional demands and pre-retirement planning. In this context, inconsistent support can be categorized as organizational stressors, which have been identified as common and challenging issues for high-performance athletes (Arnold et al., 2017b). The persistence of unaddressed organizational stressors can lead to various adverse outcomes, including burnout, injury, negative emotions and affect, and significant impacts on health and well-being (e.g., Tabei et al., 2012; Cross et al., 2016; Arnold et al., 2017a). These consequences can, over time, profoundly affect athletes’ transition out of sport, necessitating increased attention and intervention from sport governing bodies and related authorities. As mentioned earlier, participants pointed out that the establishment of pre-retirement planning necessitated support, particularly from sports governing bodies. In addition, several participants (Olympians 1, 2, and 8) expressed frustration over the lack of organizational support. This feedback should be integrated into the Conceptual Model of Adaptation to Career Transition (Lavallee et al., 2014), particularly under “Factors Related to Adaptation to Career Transition” (e.g., the absence of organizational support). Other participants also highlighted that the lack of a clear point of contact for support made it challenging to consider establishing pre-retirement plans, which should be also integrated into the model.

While none of them had put pre-retirement planning into action during their athletic careers, it was notable that some British judokas did benefit significantly from their agencies in terms of developing a career plan. These observations highlight the pressing need for organizational support that maintain a balance between pursuing excellence and facilitating preparation for post-athletic lives, emphasizing pre-retirement planning. While earlier studies have documented the existence of structured support programs worldwide, which include pre-retirement planning provisions (e.g., Hong and Coffee, 2018; Torregrossa et al., 2020), there is still a compelling case for more rigorous implementation. Such insights should also be incorporated into the Conceptual Model of Adaptation to Career Transition (Lavallee et al., 2014), especially under “Available Resources for Adaptation to Career Transition” (e.g., well-structured organizational support). In addition, it is crucial to emphasize that athletes must be aware of the importance of establishing pre-retirement planning to sufficiently prepare for their post-sport lives and ensure long-term well-being and quality of life. It is also important to widely publicize successful examples of pre-retirement planning to help athletes understand its significance. Mentorship can be an effective approach in this context. Park and Lavallee (2015) proposed that practitioners could offer mentorship support from individuals who have successfully navigated athletic career transitions and adjusted to life after sports, which is a crucial consideration in the provision of support.

From the perspective of organizational support, participants acknowledged the significance of such support in fueling their athletic aspirations throughout their careers. Nevertheless, they also felt that when this support is removed by the politics and agendas of sports governing bodies, it can have a discouraging effect on their ambitions. Every participant received financial support from their respective sport governing bodies during their peak performance times, especially as they prepared for the Olympics. This financial assistance was instrumental in advancing their careers. Yet, it is noteworthy to mention that the British judokas confronted substantial funding cuts due to the sport governing body’s decision to shift training to a central training center. All of them declined this transition, leading to shared negative experiences. Importantly, one participant emphasized that such politics and agendas played a pivotal role in their decision to retire. This emphasized the potential adverse impacts of unfavorable politics and agendas, which can profoundly affect an athlete’s performance and might even prompt early career termination. These insights should be incorporated into the Conceptual Model of Adaptation to Career Transition (Lavallee et al., 2014), specifically under “Causes of Career Termination” (e.g., shifts in politics and agendas). As previously noted, changes in politics and agendas within sport governing bodies that negatively impact athletes’ careers and performances can also be categorized as organizational stressors, particularly concerning logistical and environmental issues (Arnold et al., 2017b). To mitigate such organizational stressors, it is critical for sport governing bodies to establish clear communication channels, involve athletes in decision-making processes, and implement well-structured support systems that address the unique needs and challenges faced by athletes.

Psychological support from sport governing bodies is considered particularly significant during the retirement phase. Only one participant (Olympian 5) had received such psychological support, and she emphasized the need for more accessible and consistent support, especially when athletes confront challenges such identity loss during retirement. The lack of such support is considered detrimental to a smooth transition. For instance, the tragic suicide of former world judo champion Craig Fallen in 2019, following his retirement (Keay, 2019), highlights the urgent need for structured and customized support for athletes transitioning out of their sports careers. Many cases of athletes struggling with post-retirement challenges, such as loss of identity and motivation, financial difficulties, job insecurity, and loss of life goals, have been extensively documented (e.g., BBC, 2018), all of which strongly point to a lack of pre-retirement planning during their athletic careers as highlighted previously. In this context both this study and prior research (e.g., Stambulova et al., 2009, 2021; Park et al., 2013; Torregrosa et al., 2015; Hong and Coffee, 2018) confirm that psychological support is critical for high-performance athletes, both during their careers and as they transition out of their sport. As highlighted by the Conceptual Model of Adaptation to Career Transition (Lavallee et al., 2014), psychological support is a cornerstone of effective organizational interventions and should be central to support services and programs.

### 4.1. Practical application

The insights gained from the present study provide a foundation for developing a path for athletes’ retirement and facilitating their quality lives post-athletics. Firstly, a proactive approach to retirement planning is critical. Sport governing bodies should initiate engaging athletes in pre-retirement planning at an early stage in their careers, focusing on holistic development that includes not only athletic but also personal and professional development. Secondly, fostering strong relationships with coaches, peers, and support staff is also crucial as they play a significant role in the athlete’s journey and can provide valuable support during the transition phase. Thirdly, the provision of consistent and structured psychological support is key to addressing challenges such as identity loss and goal loss. Mentorship programs involving retired athletes who have successfully navigated the transition can provide valuable insights and support. In addition, promoting successful examples of pre-retirement planning and post-athletic career development can serve as a motivation and guide for athletes. Lastly, sport governing bodies should establish clear communication channels, involve athletes in decision-making processes, and address organizational stressors that can negatively impact the transition. Providing guidance on financial management, career development, and setting life goals post-retirement are also significant components of the support program. Thus, a holistic, structured, and proactive approach to retirement planning that addresses the unique needs and challenges faced by athletes is essential to facilitate a smoother transition into post-athletic life and ensure their long-term well-being and quality of life.

### 4.2. Limitations of the study and future research direction

While this study offers meaningful contributions, it also has limitations. It was not initially designed to explore differences in experiences based on cultural backgrounds. However, data analysis did uncover some such differences to a degree. A deeper exploration of these cultural nuances was beyond the study’s scope, but future research could examine the experiences of Olympians, factoring in their cultural backgrounds and their countries’ sports systems, both of which can significantly influence their careers. Although judo is one of the representative Olympic sports, there is a dearth of research on judokas’ experiences. Thus, examining their experiences was valuable. Still, future research could expand to include athletes from other sports, especially team sports, to offer a more comprehensive perspective on the pursuit of Olympic excellence and the subsequent transition out of sport. In this respect, studying sports less prominent in public discourse or newly introduced to the Olympics might also provide fresh insights.

## 5. Conclusion

The present study explored the challenges faced by retired Olympian judokas including issues of identity and goal loss, mental health challenges, and a lack of adequate support from sport governing bodies during their transition. It also highlighted the adverse effects of insufficient pre-retirement planning. Despite these challenges, the resilience of athletes in adapting to post-athletic life was evident. The findings emphasize the need for a proactive, structured, and holistic approach to athletes’ retirement planning, involving financial guidance, psychological support, mentorship, and decision-making involvement from an early stage in their careers. Although the study provides valuable insights into judokas’ experiences, it also indicates the necessity for broader research covering a diverse range of sports and cultural backgrounds. In conclusion, this study emphasizes the importance of a holistic strategy in preparing for retirement to ease the shift into life after sports, thereby ensuring sustained mental and physical health and overall life satisfaction for retired athletes.

## Data availability statement

The original contributions presented in this study are included in the article/supplementary material, further inquiries can be directed to the corresponding author.

## Ethics statement

The studies involving humans were approved by the General University Ethics Panel (GUEP) of the University of Stirling. The studies were conducted in accordance with the local legislation and institutional requirements. The participants provided their written informed consent to participate in this study. Written informed consent was obtained from all participants for the publication of any potentially identifiable images or data included in this article.

## Author contributions

HJH: Conceptualization, Methodology, Formal analysis, Investigation, Data curation, Writing—original draft, Writing—review and editing, Visualization, Project administration. SHH: Conceptualization, Methodology, Formal analysis, Investigation, Data curation, Writing—review and editing.
